# Association of Shift Work and Serum Vitamin D Levels with Excessive Daytime Sleepiness, Anxiety Symptoms, and Depressive Symptoms in Female Nurses

**DOI:** 10.3390/metabo16100786

**Published:** 2026-10-16

**Authors:** İbrahim Safa Karcı, Kamile Marakoğlu, Fatma Hümeyra Yerlikaya, Zeynep Ebru Şener

**Affiliations:** 1 Department of Family Medicine, Faculty of Medicine, Selçuk University, 42130 Konya, Türkiye; ibrahimsafa.karci@saglik.gov.tr (İ.S.K.); zeynep.sener@selcuk.edu.tr (Z.E.Ş.); 2 Department of Medical Biochemistry, Faculty of Medicine, Selçuk University, 42130 Konya, Türkiye; fhumeyray@selcuk.edu.tr

**Keywords:** shift work, vitamin D, 25-hydroxyvitamin D, excessive daytime sleepiness, anxiety, depression, nurses

## Abstract

Background/Objectives: This study evaluated the associations of shift work with serum vitamin D levels, excessive daytime sleepiness (EDS), and symptoms of anxiety and depression among female nurses working at a university hospital. Methods: This was an analytical cross-sectional study. Sociodemographic and occupational characteristics were collected via a structured questionnaire. EDS was assessed using the Epworth Sleepiness Scale (ESS), and symptoms of anxiety and depression were assessed using the Hospital Anxiety and Depression Scale (HADS). Serum 25-hydroxyvitamin D [25(OH)D] levels were measured by electrochemiluminescence (Elecsys Vitamin D total II, cobas e 801). Deficiency was defined as <20 ng/mL. Results: Mean age was 32.4 ± 6.8 years; 44.9% worked night shifts. Mean 25(OH)D level was 13.16 ± 7.87 ng/mL, with deficiency in 84.3% and insufficiency in 13.5%. EDS was present in 30.8%, anxiety symptoms in 26.9%, and depressive symptoms in 46.2%. Vitamin D levels were lower in night-shift nurses, though not significantly (*p* = 0.056). In univariable logistic regression analysis, night-shift work was associated with vitamin D deficiency (OR = 2.053; 95% CI: 1.068–3.948; *p* = 0.031), but this association was not significant after adjustment (aOR = 1.136; 95% CI: 0.541–2.389; *p* = 0.736). EDS was associated with anxiety symptoms (aOR = 2.795; 95% CI: 1.553–5.031; *p* = 0.001) and depressive symptoms (aOR = 1.887; 95% CI: 1.077–3.306; *p* = 0.027). Anxiety and depressive symptoms were associated with each other (aOR = 7.332 and 7.285; *p* < 0.001). Conclusions: A high prevalence of vitamin D deficiency, EDS, anxiety symptoms and depressive symptoms was observed among female nurses. Vitamin D status was not significantly associated with EDS, anxiety symptoms, or depressive symptoms, whereas EDS was associated with both anxiety and depressive symptoms.

## 1. Introduction

With the technological and economic developments of the 21st century, shift work has become widespread worldwide; in sectors such as healthcare, security, and industry—which require round-the-clock service—it has become a mandatory form of work. In industrialized countries, approximately one-fourth of the workforce engages in shift work; this proportion rose from 17% in Europe in 2005 to 21% in 2015, while in the United States, it was reported to be 38%. In terms of sectoral distribution, shift work is most common in the healthcare sector, followed by transportation and manufacturing [1].

While this schedule is essential for the continuity of health care services, it can negatively affect the physiological, psychological, and social well-being of workers. Shift work fundamentally disrupts the circadian rhythm, leading to irregularities in many physiological functions, particularly body temperature, hormone secretion, and metabolic processes. This effect is particularly pronounced during night shifts and has been linked to sleep disorders, excessive daytime sleepiness, psychological symptoms, and metabolic changes [2,3].

Disruptions in sleep patterns among shift workers are so common that they have led to the identification of a distinct clinical entity over time. Shift Work Sleep Disorder (SWSD) is listed as a separate diagnostic category in the Diagnostic and Statistical Manual of Mental Disorders, Fifth Edition (DSM-5), International Classification of Diseases, Tenth Revision (ICD-10), and International Classification of Sleep Disorders, Third Edition (ICSD-3). According to the DSM-5, the disorder develops as a result of a mismatch between work schedules and the biological sleep–wake rhythm; it is most commonly observed in individuals working night, early morning, and rotating shifts, and it typically manifests as difficulty falling asleep, inability to maintain sleep, or excessive daytime sleepiness, thereby negatively affecting the individual’s quality of life and functioning [4]. Reported to have a general prevalence of 26.5% [5], the most prominent daytime manifestation of this disorder is Excessive Daytime Sleepiness (EDS) occurring during work hours. EDS is a major cause of workplace accidents and occupational errors and represents a critical risk factor for both worker health and patient safety [6,7].

Another potential consequence of night shifts relates to vitamin D metabolism. Since the primary source of vitamin D is its synthesis in the skin through exposure to sunlight, work schedules and the amount of time spent in daylight may influence serum vitamin D levels [8]. The tendency of night workers to sleep during daytime hours may reduce their exposure to daylight and the resulting synthesis, thereby lowering vitamin D levels. Indeed, vitamin D deficiency and insufficiency are commonly observed among healthcare workers, particularly nurses, and it has been reported that this condition may be associated with shift work schedules [9,10].

Vitamin D is a hormone that plays a role not only in calcium balance and skeletal health but also in nervous system function. While it maintains calcium homeostasis through its effects on the intestines, kidneys, bones, and parathyroid glands, it is also associated with mental health functions due to its effects on nervous system development, neurotransmitter synthesis, and neuroinflammation [11]. Furthermore, vitamin D deficiency has been linked to an increased risk of autoimmune diseases; it has been shown that vitamin D3 supplementation reduces the incidence of autoimmune diseases and upper respiratory tract infections and leads to a significant increase in regulatory T (Treg) cells [12]. Given the regulatory role of vitamin D receptors in the brain—including the synthesis of neurotransmitters such as dopamine and norepinephrine and their influence on the hypothalamic–pituitary–adrenal (HPA) axis—it has been proposed that there is a strong link between vitamin D deficiency and the pathophysiology of anxiety and depression [13,14].

Consistent with this relationship, studies show that the prevalence of anxiety symptoms and depressive symptoms is higher among shift workers than among day workers [15]. Shift work has been associated with sleep problems and psychological symptoms among nurses, suggesting that these conditions should not be assessed independently of one another [16,17].

Although shift work has often been examined in relation to individual health outcomes, there are only a limited number of studies that have simultaneously examined vitamin D levels, excessive daytime sleepiness, and symptoms of anxiety and depression in the same sample. Accordingly, this study aimed to evaluate the associations of shift work with serum vitamin D levels, excessive daytime sleepiness, and symptoms of anxiety and depression among female nurses working at a university hospital.

## 2. Materials and Methods

### 2.1. Type and Purpose of the Study

This study was designed as an analytical cross-sectional study. The study was designed and reported in accordance with the STROBE (Strengthening the Reporting of Observational Studies in Epidemiology) statement for cross-sectional studies. The aim of this study was to evaluate the associations of shift work with serum vitamin D levels, excessive daytime sleepiness, and symptoms of anxiety and depression among female nurses working at Selçuk University Faculty of Medicine Hospital.

### 2.2. Setting, Population, Sample, and Participants

The study was conducted at a university hospital between 1 May 2025, and 31 July 2025, following approval by the Selçuk University Faculty of Medicine Local Ethics Committee under Decision No. 2025/51 dated 28 January 2025. During the study period, there were 767 nurses employed at the hospital, 553 of whom were women; these female nurses constituted the study population.

**Figure 1:**
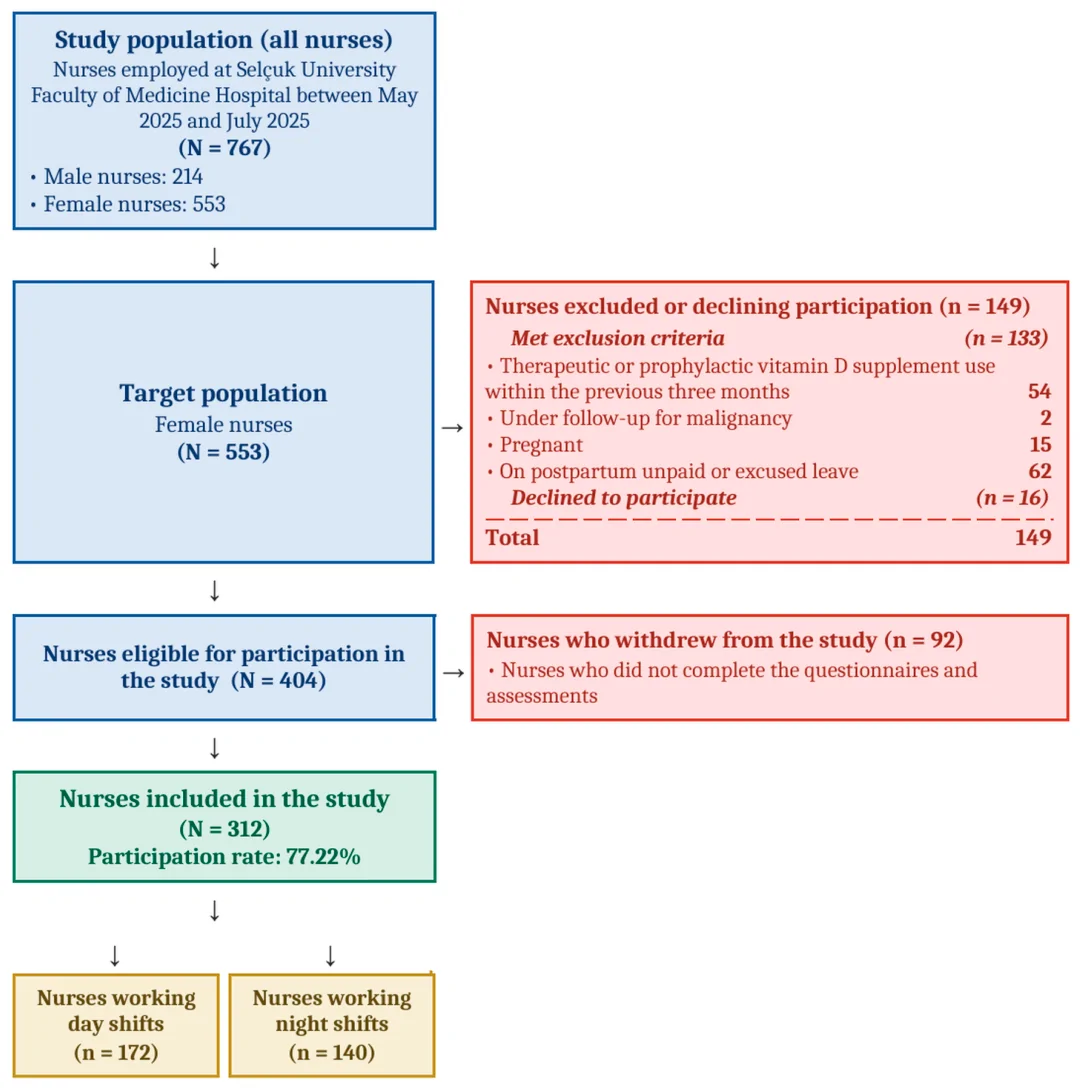
Participant enrollment flowchart.

Female nurses aged 18 and older who volunteered to participate in the study were included. The exclusion criteria were therapeutic or prophylactic vitamin D use within the previous three months, follow-up for malignancy, pregnancy, and postpartum unpaid or excused leave. Of the 553 female nurses, 133 met at least one exclusion criterion and 16 declined to participate, leaving 404 nurses eligible for the study. Following the withdrawal of 92 nurses who either refused to or were unable to complete the questionnaire and assessments during the data collection process, the study was completed with 312 female nurses. The participation rate was 77.22% (312/404). The process of including and excluding participants from the study is shown in Figure 1.

At the hospital where the study was conducted, the shift system was organized into two types: day shifts (8:00 a.m.–4:00 p.m.) and night shifts (4:00 p.m.–8:00 a.m.). Shift type was determined according to the participants’ fixed work schedule during the previous three months, with no shift rotation during this period. Of the nurses included in the study, 172 worked day shifts and 140 worked night shifts.

The required sample size was determined a priori using a power analysis. To determine whether there were statistically significant differences in vitamin D levels between day and night shifts at Selçuk University Faculty of Medicine Hospital, the study was determined to require at least 210 participants (105 in each group) for an independent-samples *t*-test, assuming a 5% significance level, 95% statistical power, and an assumed effect size (Cohen’s d) of 0.50. The power analysis was performed using the “pwr” package in R version 4.2.1. The final sample of 312 participants exceeded the minimum sample size estimated a priori; with the achieved group sizes (172 day-shift and 140 night-shift nurses), the study had 95% power to detect a between-group difference in serum vitamin D levels corresponding to an effect size as small as d ≈ 0.41.

### 2.3. Data Collection and Scales Used

A questionnaire consisting of three sections was administered via face-to-face interviews to nurses who provided informed consent through a volunteer consent form. The first section included sociodemographic information, the second section included the ESS, and the third section included the HADS. Venous blood samples were then collected from the participants and analyzed in the same institution’s laboratory (see Section 2.4).

Sociodemographic data were collected using a questionnaire prepared by the researcher. The questionnaire inquired about gender, age, self-reported height and self-reported weight (used to calculate Body Mass Index [BMI]), marital status, lifestyle, years in the profession, department of employment and length of service in that department, tobacco and alcohol use, comorbidities, medications used, previous vitamin D supplement use, clothing style (seasonal clothing, allowing greater skin exposure depending on the season, or body-covering clothing), and shift types over the past three months. Previous vitamin D supplement use was assessed, and participants who had used vitamin D supplements within the previous three months were excluded from the study. Dietary vitamin D and calcium intake, sunscreen use, and quantitative sunlight exposure were not systematically assessed.

The Epworth Sleepiness Scale is an eight-item measure that assesses EDS [18]. Each item is scored on a scale of 0–3; the total score ranges from 0 to 24, and scores above 10 indicate the presence of excessive daytime sleepiness. The validity and reliability study of the Turkish version of the scale was conducted by Ağargün et al. (1999) [19].

The Hospital Anxiety and Depression Scale consists of a total of 14 items, seven of which assess anxiety symptoms and seven of which assess depressive symptoms [20]. In a validity and reliability study conducted in Turkey, the cutoff score was determined to be 10 for the anxiety subscale and 7 for the depression subscale [21].

### 2.4. Serum 25(OH)D Analysis

Approximately 5 mL of venous blood was collected from each participant into a gel-containing serum tube. After allowing the samples to clot at room temperature for 30 min, they were centrifuged at 3000 rpm for 10 min, and the resulting serum was stored at −20 °C until the day of analysis. Samples were thawed once before analysis and were not subjected to repeated freeze–thaw cycles. Serum 25-hydroxyvitamin D [25(OH)D] levels were measured using the Elecsys Vitamin D total II assay developed by Roche Diagnostics on a cobas e 801 immunochemical analyzer via the electrochemiluminescence method. The method, with a measurement range of 3–100 ng/mL, is standardized against the ID-LC-MS/MS 25(OH)D reference method. According to the manufacturer, the repeatability and intermediate precision coefficients of variation ranged from 1.1% to 8.9% and from 2.2% to 10.8%, respectively. The results were interpreted based on the Endocrine Society Clinical Practice Guidelines; accordingly, levels <20 ng/mL were classified as deficiency, 20–29.99 ng/mL as insufficiency, and ≥30 ng/mL as sufficiency [22]. All blood parameters were analyzed at the Department of Biochemistry laboratory at Selçuk University Faculty of Medicine; daily, monthly, and quarterly maintenance and calibration procedures for the instruments were performed regularly to ensure the accuracy and reliability of the measurements.

### 2.5. Statistical Analysis

Statistical analyses were performed using R 4.2.1 (The R Foundation for Statistical Computing, Vienna, Austria). Data normality was assessed using the Shapiro–Wilk test and Q-Q plots, and homogeneity of variances was evaluated using the Levene test. Numerical variables are presented as mean ± standard deviation or median (interquartile range [IQR]), and categorical variables are presented as frequency (*n*) and percentage. For two-group comparisons, the independent samples *t*-test, Welch’s *t*-test, or the Mann–Whitney U test were used. Effect sizes were reported for the statistical tests performed. For independent-samples *t*-tests, Cohen’s d was used, whereas for chi-square tests, Phi (φ) or Cramér’s V was used, as appropriate. The distribution of categorical variables across groups was compared using the Pearson chi-square test, the Yates-corrected chi-square test, or the Fisher exact test.

Relationships between continuous variables were examined using the Pearson or Spearman correlation coefficient, and the coefficients were interpreted as indicating a negligible (<0.10), low (0.10–0.39), moderate (0.40–0.69), high (0.70–0.89), or very high (0.90–1.00) level of association based on their absolute values. Factors associated with vitamin D deficiency, excessive daytime sleepiness, anxiety symptoms, and depressive symptoms were evaluated using univariable and multivariable binary logistic regression analyses. Covariates for the multivariable logistic regression models were selected based on associations observed in univariable analyses, clinical and biological relevance, findings reported in previous studies, and potential overlap or correlations among candidate variables. In models including shift type, variables that differed significantly between day- and night-shift nurses were also evaluated as potential confounders based on their clinical relevance to the outcome. For vitamin D deficiency, age, marital status, presence of a child at home, shift type, clothing style, and previous vitamin D supplement use were included in the multivariable model. Marital status was categorized as married and not married; the not-married category included single, separated/divorced, and widowed participants. Professional experience was not included in this model because of its strong correlation with age. Work unit was also not included because administrative units were staffed exclusively by day-shift nurses, resulting in substantial overlap with shift type. For excessive daytime sleepiness, shift type, professional experience, anxiety symptoms, depressive symptoms, and BMI were included in the multivariable model. For anxiety symptoms, EDS and depressive symptoms were included in the multivariable model. For depressive symptoms, excessive daytime sleepiness, anxiety symptoms, shift type, and BMI were included in the multivariable model. Pack-years were not included in the multivariable models because this variable was available only for participants with a history of smoking and its inclusion would have substantially reduced the analytic sample. Because anxiety and depressive symptoms represent related psychological dimensions, the depression subscale (HADS-D) was entered as a covariate in the anxiety model and the anxiety subscale (HADS-A) in the depression model to account for their statistical association when estimating the adjusted relationships with each outcome. Multicollinearity was assessed using variance inflation factors (VIFs), and model fit was evaluated using the Hosmer–Lemeshow goodness-of-fit test. Results were presented as odds ratios (ORs) and adjusted odds ratios (aORs) with 95% confidence intervals (CIs), and *p* < 0.05 was considered statistically significant. The comparison of vitamin D status between day-shift and night-shift nurses was considered the primary analysis; all other analyses were considered exploratory.

## 3. Results

The study included 312 female nurses working in the inpatient wards, outpatient clinics, emergency departments, administrative units, and intensive care units of Selçuk University Faculty of Medicine. The nurses’ ages ranged from 23.0 to 53.0 years, with a mean age of 32.42 ± 6.76 years. Of the nurses, 55.1% (*n* = 172) worked day shifts, and 44.9% (*n* = 140) worked night shifts. Among the 92 eligible nurses who did not complete the assessments, 49 (53.3%) worked night shifts and 43 (46.7%) worked day shifts. Shift distribution did not differ significantly between participants who completed the study and those who did not (Pearson χ^2^ = 2.008, *p* = 0.156).

Sociodemographic and clinical characteristics by shift type are presented in Table 1. The mean age of nurses working the day shift was higher than that of those working the night shift (34.21 ± 6.97 vs. 30.22 ± 5.81 years; *p* < 0.001). Similarly, the mean BMI was 25.77 ± 4.87 kg/m^2^ for those working the day shift and 24.26 ± 4.21 kg/m^2^ for those working the night shift (*p* = 0.004). While 76.7% (*n* = 132) of those working the day shift were married, this rate was 43.6% (*n* = 61) among those working the night shift; while the proportions of those who were single, divorced, or widowed were 23.3% (*n* = 40) and 56.4% (*n* = 79), respectively (*p* < 0.001). The distribution of work units also differed by shift type (*p* < 0.001); specifically, the proportion working in intensive care/emergency units was 48.6% (*n* = 68) on the night shift and 23.3% (*n* = 40) on the day shift. The duration of professional experience was longer among those working the day shift (12.20 ± 6.84 years vs. 7.74 ± 5.76 years; *p* < 0.001). The prevalence of chronic disease was 40.1% (*n* = 69) among those working the day shift and 19.3% (*n* = 27) among those working the night shift; regular medication use was 30.2% (*n* = 52) and 12.9% (*n* = 18), respectively (*p* < 0.001 for both). Cigarette smoking was 28.5% (*n* = 49) during the day shift and 35.7% (*n* = 50) during the night shift; no significant difference was found between the groups (*p* = 0.173).

The average serum 25(OH)D level among nurses was 13.16 ± 7.87 ng/mL, and vitamin D deficiency was detected in 84.3% of them. The prevalence of EDS, anxiety symptoms, and depressive symptoms was 30.8%, 26.9%, and 46.2%, respectively. Vitamin D deficiency was more common among night-shift workers than among day-shift workers (89.3% vs. 80.2%; *p* = 0.042), but the difference in mean 25(OH)D levels was not statistically significant (*p* = 0.056). While ESS and HADS-A scores did not differ by shift type (*p* = 0.861 and *p* = 0.395, respectively), the HADS-D score was higher among night-shift workers (7.67 ± 4.10 vs. 6.72 ± 4.14; *p* = 0.042); the frequency of depressive symptoms did not differ significantly across shifts (*p* = 0.219) (Table 2).

Vitamin D deficiency was significantly associated with younger age (*p* < 0.001), marital status (*p* = 0.002), not having children at home (*p* = 0.004), shorter work experience (*p* < 0.001), and night shift work (*p* = 0.042), and previous vitamin D supplement use (*p* = 0.046). No significant differences were found in terms of BMI (*p* = 0.999), clothing style (*p* = 0.883), anxiety symptoms (*p* = 0.808), and depressive symptoms (*p* = 0.368) (Table 3).

No significant differences were found in anxiety symptoms with respect to age, professional experience, shift type, BMI, smoking status, pack-years, or vitamin D deficiency (all *p* > 0.05). No significant differences were found regarding depressive symptoms in terms of age (*p* = 0.258), professional experience (*p* = 0.614), shift type (*p* = 0.219), BMI (*p* = 0.393), smoking status (*p* = 0.573), or vitamin D deficiency (*p* = 0.368). The pack-years value, however, was higher among those without depressive symptoms (7.5 (9.0) vs. 5.0 (9.0); *p* = 0.022) (Table 4).

No significant correlation was found between serum 25(OH)D levels and ESS, HADS-A, or HADS-D scores (all *p* > 0.05). The ESS score showed a positive correlation with both the HADS-A score (r = 0.334; *p* < 0.001) and the HADS-D score (r = 0.268; *p* < 0.001). A stronger positive correlation was found between HADS-A and HADS-D scores (r = 0.568; *p* < 0.001) (Table 5).

In a univariable logistic regression analysis, night-shift workers had approximately twofold higher odds of vitamin D deficiency than day-shift workers (OR = 2.053; 95% CI: 1.068–3.948; *p* = 0.031). However, this association was no longer statistically significant in the multivariable analysis (aOR = 1.136; 95% CI: 0.541–2.389; *p* = 0.736). It was determined that each one-year increase in age was associated with a decrease in the odds of vitamin D deficiency (OR = 0.902; 95% CI: 0.864–0.941; *p* < 0.001), and this association remained significant after adjustment (aOR = 0.915; 95% CI: 0.873–0.959; *p* < 0.001). In addition, the odds of vitamin D deficiency were higher among not married participants compared to married participants (OR = 3.195; 95% CI: 1.489–6.856; *p* = 0.003) and among those without children at home compared to those with children at home (OR = 2.760; 95% CI: 1.419–5.368; *p* = 0.003); however, these associations were not statistically significant in the multivariable analysis (aOR = 1.654; 95% CI: 0.605–4.523; *p* = 0.327 and aOR = 1.136; 95% CI: 0.451–2.861; *p* = 0.787, respectively).

In the logistic regression analysis conducted for excessive daytime sleepiness, the presence of anxiety symptoms (OR = 3.723; 95% CI: 2.195–6.314; *p* < 0.001) and the presence of depressive symptoms (OR = 2.793; 95% CI: 1.697–4.595; *p* < 0.001) were associated with higher odds of EDS. Both associations remained statistically significant in the multivariable analysis (anxiety symptoms: aOR = 2.795; 95% CI: 1.553–5.031; *p* = 0.001; depressive symptoms: aOR = 1.887; 95% CI: 1.077–3.306; *p* = 0.027). In the analysis of anxiety symptoms, the presence of EDS (OR = 3.723; 95% CI: 2.195–6.314; *p* < 0.001) and the presence of depressive symptoms (OR = 8.500; 95% CI: 4.618–15.648; *p* < 0.001) were found to be significant. These associations also remained significant in the multivariable analysis (EDS: aOR = 2.807; 95% CI: 1.576–5.001; *p* < 0.001; depressive symptoms: aOR = 7.332; 95% CI: 3.937–13.657; *p* < 0.001).

In the analysis of depressive symptoms, EDS was associated with higher odds of depressive symptoms (OR = 2.793; 95% CI: 1.697–4.595; *p* < 0.001) An association was also observed between anxiety and depressive symptoms (OR = 8.500; 95% CI: 4.618–15.648; *p* < 0.001). These associations remained statistically significant in the multivariable analysis (EDS: aOR = 1.879; 95% CI: 1.081–3.266; *p* = 0.025; anxiety symptoms: aOR = 7.285; 95% CI: 3.906–13.587; *p* < 0.001). While working the night shift was not found to be significant (OR = 1.324; 95% CI: 0.846–2.073; *p* = 0.219), it also remained non-significant after adjustment (aOR = 1.300; 95% CI: 0.784–2.158; *p* = 0.310). It was found that the odds of depressive symptoms decreased as pack-years increased (OR = 0.932; 95% CI: 0.871–0.998; *p* = 0.044) (Table 6). The Hosmer–Lemeshow goodness-of-fit tests were non-significant for all multivariable models (all *p* > 0.05), and the maximum VIF values ranged from 1.06 to 3.29, suggesting no problematic multicollinearity.

## 4. Discussion

This study found that the vast majority of female nurses had deficient or insufficient vitamin D levels. The mean serum vitamin D level among participants was 13.16 ± 7.87 ng/mL, and 84.3% had vitamin D deficiency. Previous studies have also reported that vitamin D deficiency is common among healthcare workers. A recent scoping review reported that the prevalence of vitamin D deficiency among healthcare workers ranged from 30% to over 90% across studies [23]. In Türkiye, Varkal et al. reported vitamin D deficiency in 88.5% of healthcare workers, 77.1% of whom were women [9]. Rizza et al. also reported low serum 25(OH)D vitamin D levels among hospital workers in Italy [24]. In a study specifically conducted among female shift-working nurses, Tang et al. reported vitamin D deficiency in 48.4%, with 91.6% having either deficient or insufficient vitamin D levels [25]. Occupational factors such as long working hours, indoor work, shift schedules, limited sunlight exposure, and lifestyle changes related to work demands may further contribute to this burden among nurses.

Among these potential occupational factors, shift patterns are particularly noteworthy in light of our study’s findings. Although serum 25(OH)D levels were found to be lower in nurses working the night shift compared to those working the day shift, the difference did not reach statistical significance (*p* = 0.056). However, in the univariable logistic regression analysis, night-shift nurses had approximately twofold higher odds of vitamin D deficiency than day-shift nurses (OR = 2.053; 95% CI: 1.068–3.948; *p* = 0.031). This association, however, was not statistically significant in the multivariable analysis (aOR = 1.136; 95% CI: 0.541–2.389; *p* = 0.736). In a 2018 study by Lehnert et al. conducted on nurses in Germany, it was reported that the rate of vitamin D insufficiency or deficiency among shift workers was approximately 24% higher than among day workers [10]. In a study by Lee and colleagues involving 844 healthcare workers in South Korea, it was found that serum vitamin D levels were significantly lower among those working night shifts [26]. Similarly, a study by Rizza and colleagues conducted among healthcare workers in Rome showed that working rotating night shifts was associated with a decrease in serum 25(OH)D levels [24]. In contrast, a 2022 study by Tang and colleagues in Taiwan, which classified healthcare workers into fixed day, evening, and rotating night shift groups, found that average vitamin D levels were similar across the groups [25]. When evaluated on a larger scale, a systematic review and meta-analysis encompassing 13 cross-sectional studies and a total of 110,287 employees showed that serum 25(OH)D levels in shift workers were significantly lower compared to non-shift workers (mean difference: −1.85 ng/mL; 95% CI: −2.49 to −1.21) [27]. However, the high heterogeneity among the studies suggests that the relationship between shift patterns and vitamin D levels may vary depending on individual and occupational characteristics.

One possible explanation for the unadjusted association between shift work and vitamin D is reduced exposure to daylight. The fact that nurses working night shifts spend part of their daytime hours sleeping may limit the time they have to benefit from sunlight. However, the biological effects of shift work may not be limited to sunlight exposure alone. A metabolomic study conducted among female nurses reported that night shifts affect the metabolic profile and that this effect may vary depending on chronotype [28]. Similarly, a recent study among healthcare workers demonstrated that night shifts are associated with metabolic changes linked to circadian rhythms and the stress response [29]. Therefore, the association between night shifts and vitamin D deficiency may be interpreted in the context of reduced daylight exposure as well as circadian, metabolic, and lifestyle changes associated with shift work.

Another finding that may be closely related to shift work is the association between age, years of professional experience, and vitamin D levels. In our study, older age and longer professional experience were associated with a lower frequency of vitamin D deficiency. Additionally, it was observed that the proportion of workers on day shifts increased as age and professional experience increased. Analyses revealed that each 1-year increase in age was associated with approximately 8.5% lower odds of vitamin D deficiency. Similarly, in a 2016 study conducted by Aykal in Antalya involving 100 operating room staff members, it was reported that serum vitamin D levels increased with advancing age and longer duration of professional experience [30]. In a study by Rizza and colleagues conducted among healthcare workers in Rome, a positive and statistically significant association was also found between age and vitamin D levels [24]. In contrast, Martelli and colleagues’ meta-analysis reported that age did not significantly explain the difference in vitamin D levels between shift workers and non-shift workers [27]. When these findings are evaluated together, the relationship between age and vitamin D in our study may not solely reflect age-related biological changes. It may also be associated with changes in work patterns and lifestyle characteristics that occur with age. Indeed, the fact that older nurses in our sample work day shifts more frequently indicates that shift patterns must also be taken into account when interpreting the relationship between age and vitamin D. However, the cross-sectional study design does not allow for determining the direction of the relationship between these variables or the underlying mechanisms.

Other sociodemographic variables associated with vitamin D deficiency were marital status and the presence of children in the home. However, these associations were not statistically significant after adjustment for potential confounders. Although Tang and colleagues’ study, which included female nurses working rotating shifts, included subgroup comparisons based on marital status, marital status was not evaluated as an independent predictor in the multivariate analysis [25]. The limited literature on the relationship between marital status, the presence of children in the household, and vitamin D makes it difficult to interpret these findings. It is conceivable that these variables may serve as sociodemographic indicators reflecting differences in daily routines, time spent outdoors, dietary habits, and other health behaviors.

Excessive daytime sleepiness was detected in approximately one-third of nurses. The prevalence of EDS reported in healthcare workers in the literature varies widely. In a 2021 study conducted by Akçay et al. in Antalya, EDS was detected in 20% of nurses [31]. In a study by Arbour and colleagues involving 639 midwives in the United States, EDS was reported in 25.7% of participants [32]. Similarly, in a large-scale national study by Terauchi and colleagues involving 9728 female nurses in Japan, this prevalence was found to be 24.6% [33]. In contrast, in a 2019 study by Alexandropoulou and colleagues involving 444 nurses working in secondary and tertiary care hospitals in Greece, 72% of participants were classified as being at high risk for EDS based on an ESS score of ≥10 [34]. In a study by Carvalho and colleagues involving 244 healthcare workers in Brazil, the mean ESS score was determined to be 8.7 ± 0.3; according to the classification used, 50.4% of the participants reported abnormal daytime sleepiness [35]. The differences in the reported rates across studies may be related to variations in sample characteristics, work environments, and shift schedules.

Although shift work can disrupt the sleep–wake cycle, daytime sleepiness may not be explained solely by the shift type. It may also be associated with factors such as sleep duration, sleep quality, individual chronotype, mental state, and work-related stress. In a study by Rotter and colleagues, the metabolic effects of night shifts were found to vary according to chronotype, with metabolic changes being more pronounced in nurses with an early chronotype, suggesting that the response to shift work may vary depending on individual circadian characteristics [28]. Accordingly, the absence of a significant difference in ESS scores by shift type in our study suggests that daytime sleepiness should be evaluated in conjunction with other individual and work-related factors, in addition to shift type.

When nurses’ mental health was assessed in addition to daytime sleepiness, our study found that anxiety symptoms among healthcare workers were at a level that could not be ignored. In a 2022 study conducted by Uz and colleagues in Turkey with 221 healthcare workers, anxiety symptoms were found in 26.24% of the participants [36]. In a study by Øyane and colleagues involving 2035 nurses in Norway, mean HADS-A scores were reported to range between 4.5 and 4.7, and the prevalence of anxiety symptoms (HADS-A > 7) was determined to be 19.1%–21.0% [16]. In a study by Roman and colleagues involving 380 nurses in Spain, the mean HADS-A scores were reported as 11.7 ± 2.6 and 11.1 ± 2.8 for nurses working fixed shifts and rotating shifts, respectively [17]. When psychological symptoms were evaluated in conjunction with daytime sleepiness, our study revealed a significant association between EDS and anxiety symptoms. Nurses experiencing EDS had approximately 2.8-fold higher adjusted odds of anxiety symptoms. In a study conducted by Chen and colleagues involving 1048 nurses in Southern China, logistic regression analysis revealed that anxiety symptoms were independently associated with EDS and that the presence of anxiety symptoms was associated with 1.65-fold higher odds of EDS (ESS ≥ 14) [37]. When these findings are evaluated together, the association between EDS and anxiety symptoms observed in our study is consistent with the literature, suggesting that the relationship between daytime sleepiness and anxiety symptoms has also been observed in other nursing populations.

In addition to anxiety symptoms, depressive symptoms were also found to be prevalent in our sample. In the present study, the mean HADS-D score among nurses was 7.14 ± 4.14, and depressive symptoms (HADS-D > 7) were detected in 46.2% of the nurses. In a study conducted by Ezin and colleagues involving 120 nurses, the mean HADS-D score was reported as 8.88 ± 4.48 [38]. In a 2022 study by Uz and colleagues involving 221 healthcare workers in Turkey, the prevalence of depressive symptoms was found to be 39.8%; it was reported that this prevalence was higher—at 51.1%—in the nurse subgroup [36]. In a study by Øyane and colleagues involving 2035 nurses in Norway, it was reported that the mean HADS-D scores ranged from 2.4 to 2.9 depending on shift type, and the prevalence of depressive symptoms ranged from 6.2% to 10.5% [16]. In a study by Alreshidi and colleagues involving 191 participants at a tertiary-care hospital in Saudi Arabia, the mean HADS-D score was found to be 8.1 ± 0.8 [15]. These findings indicate that the levels of depressive symptoms observed in our study are similar to those reported among healthcare workers and nurses in Turkey; however, the prevalence of depressive symptoms reported in Scandinavian countries is considerably lower. When depressive symptoms were evaluated in terms of shift patterns, our study found that HADS-D scores among nurses working night shifts were significantly higher than those of nurses working day shifts. In a study conducted by Ezin and colleagues involving 120 nurses, no significant difference was found between shift status and HADS-Depression scores [38]. In contrast, a study by Dai and colleagues involving 865 nurses in China reported that HADS-D scores among nurses working the night shift (8.72 ± 3.31) were statistically significantly higher than those of day shift nurses (7.94 ± 3.77) [39]. In a study conducted by Alreshidi and Rayani with 191 healthcare workers at a tertiary care hospital, working the night shift was associated with poorer sleep quality and more depressive symptoms [15]. Although the relationship between shift type and depressive symptoms has been evaluated in the literature using different samples and measurement tools, a significant portion of the existing studies suggest an association between night-shift work and depressive symptoms. The finding in our study that HADS-D scores were higher among nurses working night shifts is consistent with this general trend. However, night-shift work was not significantly associated with depressive symptoms in the multivariable analysis (aOR = 1.300; 95% CI: 0.784–2.158; *p* = 0.310).

It is thought that disruptions in the circadian rhythm and accompanying metabolic changes may play a role in the association between night shifts and depressive symptoms. A metabolomic study conducted among healthcare workers reported that night shifts are associated with changes in metabolic pathways related to circadian regulation and the stress response [29]. These findings suggest that circadian and metabolic processes may also play a role in the higher HADS-D scores observed in nurses working night shifts. However, since these mechanisms were not directly assessed in our study, no causal or mechanistic conclusions can be drawn regarding this matter.

One of the notable findings of our study is the relationship between EDS and symptoms of anxiety and depression. It was found that symptoms of anxiety and depression were more common among nurses experiencing excessive daytime sleepiness. In the univariable logistic regression analysis, the odds of EDS were approximately 3.7-fold higher among nurses with anxiety symptoms and 2.8-fold higher among those with depressive symptoms. These associations remained statistically significant in the multivariable analysis (aOR = 2.795 and aOR = 1.887, respectively). These findings suggest that EDS may be closely related to symptoms of anxiety and depression. Similarly, in a study by Chen and colleagues involving 1048 nurses in China, both depressive and anxiety symptoms were independently associated with EDS [37]. Additionally, anxiety and depressive symptoms were associated with each other in both the univariable analysis (OR = 8.500; *p* < 0.001) and the multivariable analyses (aOR = 7.332 in the anxiety symptoms model and aOR = 7.285 in the depressive symptoms model; both *p* < 0.001). A study conducted by Uz and colleagues involving 221 healthcare workers reported a strong, positive correlation between HADS-D and HADS-A scores [36]. In a study conducted by Roman and colleagues in Spain involving 380 nurses, it was reported that nurses working rotating shifts had both lower sleep quality—as assessed by the Pittsburgh Sleep Quality Index—and higher HADS-D scores [17]. These findings suggest that there may be a multidimensional and interrelated structure among shift work, daytime sleepiness, and psychological symptoms. Given the cross-sectional design and contemporaneous assessment of HADS-A and HADS-D, the observed association between anxiety and depressive symptoms does not establish directionality or causality and may partly reflect shared psychological variance between the two related symptom dimensions. Similarly, the temporal direction of the associations between EDS and psychological symptoms cannot be established, and these relationships may be bidirectional.

Despite these clear associations between sleep and psychological symptoms, our study did not find a significant association between serum vitamin D levels and EDS, HADS-A, and HADS-D scores. Although vitamin D has been hypothesized to be associated with sleep and mental health due to its effects on nervous system functions, neurotransmitter synthesis, and neuroinflammatory processes, this association could not be demonstrated in our study. However, there are studies in the literature reporting an association between low serum vitamin D levels and depression. In an umbrella meta-analysis published by Musazadeh et al. in 2023, a combined evaluation of data from cohort studies reported that low serum vitamin D levels were associated with higher odds of depression (OR = 1.60; 95% CI: 1.08–2.36); however, high heterogeneity among the studies was noted [40]. Similarly, in a systematic review and meta-analysis by Karim et al. in 2026, which included 15 studies and 49,931 participants, low vitamin D levels were also found to be associated with higher odds of depression (OR = 1.45; 95% CI: 1.36–1.56) [41]. Unlike these studies, our study did not find a significant association between vitamin D levels and depressive symptoms. This discrepancy may be related to sample-specific characteristics, such as the fact that our study was conducted on a more homogeneous sample consisting solely of female nurses and that the vast majority of participants had vitamin D deficiency.

One possible reason why our study failed to demonstrate an association between vitamin D and sleep-related and psychological symptoms may be the presence of vitamin D deficiency in a very large proportion of the sample (84.3%). The fact that vitamin D levels were largely clustered in the deficiency range may have led to a smaller comparison group with adequate vitamin D levels, reduced biological contrast between the groups, and limited the statistical power to identify potential associations. Furthermore, EDS, anxiety symptoms, and depressive symptoms are multifactorial outcomes, and factors such as shift patterns, sleep duration and quality, chronotype, work intensity, job stress, and other psychosocial factors may be more closely related to them than vitamin D levels. Therefore, the lack of association observed in our study should be interpreted not as evidence that there is no relationship between vitamin D and sleep-related or psychological symptoms, but rather as an indication that such a relationship could not be demonstrated within the constraints of the current sample and study design. Accordingly, these findings should be interpreted within the context of this study population and should not be generalized to populations with a broader distribution of vitamin D levels or a higher proportion of individuals with adequate vitamin D status.

The cross-sectional and single-center nature of the study limits the ability to establish causal relationships and to generalize the findings to all nurses. Moreover, 92 eligible nurses did not complete the questionnaires or assessments; although their shift distribution was similar to that of the participants included in the final analysis, a comprehensive comparison of their other demographic and clinical characteristics was not possible because of incomplete data. Therefore, potential selection bias due to non-participation cannot be excluded; depending on whether non-completion was related to workload or symptom burden, the prevalence of the outcomes may have been under- or overestimated. Such differential participation may also have influenced the observed associations, although the direction and magnitude of this potential bias remain uncertain. Consequently, caution is warranted when generalizing the findings to the full eligible nursing population. Additionally, the relatively small size of the non-deficient vitamin D group (*n* = 49) resulted in an events-per-variable ratio of approximately 8 in the vitamin D deficiency model, slightly below the conventional threshold of 10. Although model fit (Hosmer–Lemeshow *p* = 0.212) and multicollinearity diagnostics (maximum VIF = 2.10) did not indicate any problems, this may have reduced the precision and stability of the adjusted estimates and limited the statistical power to detect associations between vitamin D status and the outcomes examined; larger studies with more balanced vitamin D status groups are warranted. Although work unit was recorded, it was not included in the adjusted vitamin D model because administrative units were staffed exclusively by day-shift nurses, resulting in substantial overlap with shift type. Quantitative sunlight exposure was not assessed, although differences in opportunities for sunlight exposure across shift schedules may have influenced serum 25(OH)D concentrations. Dietary vitamin D intake was also not systematically evaluated; although cutaneous synthesis is an important source of vitamin D, dietary differences may still contribute to variation in vitamin D status. Furthermore, sleep duration and quality were not comprehensively assessed and may have influenced excessive daytime sleepiness and its associations with anxiety and depressive symptoms. Although participants were classified according to shift type, this classification did not account for individual chronotype, which may influence adaptation to work schedules and sleep-related outcomes. Finally, although restricting the study population to nurses reduced occupational heterogeneity, differences in workload and occupational stress across clinical units may still have influenced psychological symptoms. These unmeasured factors may have contributed to residual confounding, and the direction and magnitude of their effects remain uncertain. In addition, the number of comparisons performed may increase the probability of chance findings, particularly for results with *p* values close to 0.05 in the exploratory analyses. As no formal correction for multiple testing was applied, marginal findings should be interpreted cautiously, with consideration of effect estimates and 95% confidence intervals rather than isolated *p* values. In addition, the robustness of the findings across alternative model specifications was not evaluated through sensitivity analyses, which should be considered when interpreting the adjusted associations. Furthermore, because all measurements were obtained between May and July, the findings reflect vitamin D status during a relatively high-sunlight period and may not represent year-round vitamin D status. Seasonal variation should therefore be considered when generalizing these findings to other times of the year. However, the inclusion of 312 female nurses, the measurement of serum 25(OH)D levels using a standardized laboratory method, and the concurrent assessment of vitamin D, daytime sleepiness, anxiety symptoms, and depressive symptoms within the same sample are the study’s strengths.

## 5. Conclusions

Vitamin D deficiency was highly prevalent among female nurses. After adjustment for potential confounders, night-shift work was not significantly associated with vitamin D deficiency, although an association was observed in the univariable analysis. Although no significant association was found between serum vitamin D levels and excessive daytime sleepiness, anxiety symptoms, or depressive symptoms, associations were observed between excessive daytime sleepiness and symptoms of anxiety and depression. These findings suggest that sleep-related and psychological symptoms should be considered together when evaluating the health and well-being of female nurses. Given the cross-sectional design, these findings reflect associations and do not establish causality or directionality; longitudinal studies are needed to clarify these relationships.

## Figures and Tables

**Table 1:** Demographic and Clinical Characteristics by Shift Type.

Variable	Total (*N* = 312)	Day Shift (*n* = 172)	Night Shift (*n* = 140)	*ES*	*p*
Age (years), mean ± SD	32.42 ± 6.76	34.21 ± 6.97	30.22 ± 5.81	**−0.616**	**<0.001 ^3^**
BMI (kg/m^2^), mean ± SD	25.09 ± 4.64	25.77 ± 4.87	24.26 ± 4.21	**−0.328**	**0.004 ^2^**
Marital status, *n* (%)				**0.403**	**<0.001 ^1^**
Married	193 (61.9)	132 (76.7)	61 (43.6)		
Single/separated/widowed	119 (38.1)	40 (23.3)	79 (56.4)		
Working unit, *n* (%)				**0.341**	**<0.001 ^1^**
Internal medicine	97 (31.1)	59 (34.3)	38 (27.1)		
Surgical medicine	82 (26.3)	48 (27.9)	34 (24.3)		
ICU/Emergency	108 (34.6)	40 (23.3)	68 (48.6)		
Administrative	25 (8.0)	25 (14.5)	0 (0.0)		
Professional experience (years), mean ± SD	10.20 ± 6.75	12.20 ± 6.84	7.74 ± 5.76	**−0.699**	**<0.001 ^3^**
Chronic disease, *n* (%)				**0.224**	**<0.001 ^1^**
No	216 (69.2)	103 (59.9)	113 (80.7)		
Yes	96 (30.8)	69 (40.1)	27 (19.3)		
Regular medication use, *n* (%)				**0.207**	**<0.001 ^1^**
No	242 (77.6)	120 (69.8)	122 (87.1)		
Yes	70 (22.4)	52 (30.2)	18 (12.9)		
Current smoking, *n* (%)				0.077	0.173 ^1^
No	213 (68.3)	123 (71.5)	90 (64.3)		
Yes	99 (31.7)	49 (28.5)	50 (35.7)		

Values are presented as mean ± SD or n (%). *p* values were obtained using ^1^ Pearson’s chi-square test, ^2^ independent-samples *t* test, and ^3^ Welch’s *t* test, as appropriate. BMI = body mass index; ICU = intensive care unit. Significant *p* values (*p* < 0.05) are shown in bold. ES: Effect Size.

**Table 2:** Vitamin D Status, Daytime Sleepiness, Anxiety Symptoms, and Depressive Symptoms According to Shift Type.

Variable	Total (*N* = 312)	Day Shift (*n* = 172)	Night Shift (*n* = 140)	*ES*	*p*
25(OH)D (ng/mL), mean ± SD	13.16 ± 7.87	13.92 ± 7.89	12.22 ± 7.77	−0.218	0.056 ^1^
Vitamin D deficiency, n (%)	263 (84.3)	138 (80.2)	125 (89.3)	**0.124**	**0.042 ^2^**
ESS score, mean ± SD	8.83 ± 4.97	8.88 ± 5.23	8.78 ± 4.64	−0.020	0.861 ^1^
Excessive daytime sleepiness, n (%)	96 (30.8)	51 (29.7)	45 (32.1)	0.027	0.635 ^3^
HADS-A score, mean ± SD	8.40 ± 4.26	8.22 ± 4.33	8.63 ± 4.18	0.097	0.395 ^1^
Anxiety symptoms, n (%)	84 (26.9)	43 (25.0)	41 (29.3)	0.048	0.396 ^3^
HADS-D score, mean ± SD	7.14 ± 4.14	6.72 ± 4.14	7.67 ± 4.10	**0.232**	**0.042 ^1^**
Depressive symptoms, n (%)	144 (46.2)	74 (43.0)	70 (50.0)	0.070	0.219 ^3^

Values are presented as mean ± SD or n (%). Vitamin D deficiency was defined as serum 25-hydroxyvitamin D [25(OH)D] < 20 ng/mL; excessive daytime sleepiness as ESS > 10; anxiety symptoms as HADS-A > 10; and depressive symptoms as HADS-D > 7. *p* values were obtained using ^1^ independent-samples *t* test and ^2^ Yates’ continuity-corrected chi-square test, and ^3^ Pearson’s chi-square test, as appropriate. ESS = Epworth Sleepiness Scale; HADS-A = Hospital Anxiety and Depression Scale–Anxiety subscale; HADS-D = Hospital Anxiety and Depression Scale–Depression subscale. Statistically significant *p* values (*p* < 0.05) are shown in bold. ES: Effect Size.

**Table 3:** Comparison of Demographic and Clinical Characteristics According to Vitamin D Deficiency Status.

Variable	Total (*N* = 312)	Deficient (*n* = 263; 84.3%)	Not Deficient (*n* = 49; 15.7%)	*ES*	*p*
Age, years, mean ± SD	32.42 ± 6.76	31.59 ± 6.32	36.86 ± 7.37	**−0.811**	**<0.001 ^3^**
Marital status, *n* (%)				**0.176**	**0.002 ^1^**
Unmarried/separated/widowed	119 (38.1)	110 (41.8)	9 (18.4)		
Married	193 (61.9)	153 (58.2)	40 (81.6)		
Living arrangement, *n* (%)				**0.174**	**0.004 ^2^**
No child at home	152 (48.7)	138 (52.5)	14 (28.6)		
Child at home	160 (51.3)	125 (47.5)	35 (71.4)		
BMI group, *n* (%)				0.002	0.999 ^2^
<25 kg/m^2^	179 (57.4)	151 (57.4)	28 (57.1)		
≥25 kg/m^2^	133 (42.6)	112 (42.6)	21 (42.9)		
Professional experience, *n* (%)				**0.224**	**<0.001 ^1^**
≤10 years	154 (49.4)	142 (54.0)	12 (24.5)		
10–20 years	128 (41.0)	100 (38.0)	28 (57.1)		
≥20 years	30 (9.6)	21 (8.0)	9 (18.4)		
Shift type, *n* (%)				**0.124**	**0.042 ^2^**
Day shift	172 (55.1)	138 (52.5)	34 (69.4)		
Night shift	140 (44.9)	125 (47.5)	15 (30.6)		
Clothing style, *n* (%)				0.017	0.883 ^2^
Seasonal clothing	159 (51.0)	135 (51.3)	24 (49.0)		
Body-covering clothing	153 (49.0)	128 (48.7)	25 (51.0)		
Previous vitamin D supplement use, *n* (%)				**0.113**	**0.046 ^1^**
No	102 (32.7)	92 (35.0)	10 (20.4)		
Yes	210 (67.3)	171 (65.0)	39 (79.6)		
Anxiety symptoms, *n* (%)				0.024	0.808 ^2^
No	228 (73.1)	191 (72.6)	37 (75.5)		
Yes	84 (26.9)	72 (27.4)	12 (24.5)		
Depressive symptoms, *n* (%)				0.060	0.368 ^2^
No	168 (53.8)	145 (55.1)	23 (46.9)		
Yes	144 (46.2)	118 (44.9)	26 (53.1)		

Values are presented as mean ± SD or n (%). Percentages are column percentages. Vitamin D deficiency was defined as serum 25-hydroxyvitamin D [25(OH)D] < 20 ng/mL. Anxiety symptoms were defined as HADS-A > 10 and depressive symptoms as HADS-D > 7. BMI = body mass index. Superscripts indicate the statistical test used: ^1^ Pearson’s chi-square test; ^2^ Yates’ continuity-corrected chi-square test; ^3^ Welch’s *t*-test. Statistically significant *p* values (*p* < 0.05) are shown in bold. ES: Effect Size.

**Table 4:** Selected Characteristics According to Anxiety Symptoms and Depressive Symptoms.

Variable	Anxiety Symptoms Absent (*n* = 228)	Anxiety Symptoms Present (*n* = 84)	*ES*	*p*	Depressive Symptoms Absent (*n* = 168)	Depressive Symptoms Present (*n* = 144)	*ES*	*p*
Age, years	32.75 ± 6.84	31.52 ± 6.32	0.182	0.156 ^1^	32.85 ± 6.91	31.92 ± 6.56	0.129	0.258 ^1^
Professional experience, years	10.36 ± 6.91	9.76 ± 6.32	0.089	0.486 ^1^	10.38 ± 6.93	9.99 ± 6.56	0.057	0.614 ^1^
Shift type, *n* (%)			0.048	0.396 ^2^			0.070	0.219 ^2^
Day	129 (56.6)	43 (51.2)			98 (58.3)	74 (51.4)		
Night	99 (43.4)	41 (48.8)			70 (41.7)	70 (48.6)		
BMI, kg/m^2^	24.96 ± 4.59	25.47 ± 4.78	−0.110	0.452 ^1^	24.85 ± 4.52	25.37 ± 4.78	−0.097	0.393 ^1^
Smoking status, *n* (%)			0.083	0.143 ^2^			0.032	0.573 ^2^
Non-smoker	161 (70.6)	52 (61.9)			117 (69.6)	96 (66.7)		
Current smoker	67 (29.4)	32 (38.1)			51 (30.4)	48 (33.3)		
Pack-years	6.5 (8.25)	5.0 (8.0)	0.185	0.423 ^3^	7.5 (9.0)	5.0 (9.0)	**0.408**	**0.022 ^3^**
Vitamin D deficiency, *n* (%)			0.024	0.808 ^2^			0.060	0.368 ^2^
No	37 (16.2)	12 (14.3)			23 (13.7)	26 (18.1)		
Yes	191 (83.8)	72 (85.7)			145 (86.3)	118 (81.9)		

Values are presented as mean ± SD, median (IQR), or n (%), as appropriate. Percentages are column percentages. Vitamin D deficiency was defined as serum 25-hydroxyvitamin D [25(OH)D] < 20 ng/mL. Anxiety symptoms were defined as HADS-A > 10 and depressive symptoms as HADS-D > 7. BMI = body mass index. Superscripts indicate the statistical test used: ^1^ independent-samples *t*-test; ^2^ Pearson’s chi-square test; ^3^ Mann–Whitney U test. Statistically significant *p* values (*p* < 0.05) are shown in bold. ES: Effect Size.

**Table 5:** Correlations Between Vitamin D, Daytime Sleepiness, Anxiety Symptoms, and Depressive Symptoms.

Variable	25(OH)D	ESS	HADS-A	HADS-D
25(OH)D	—			
ESS	−0.007 (0.907)	—		
HADS-A	−0.001 (0.993)	**0.334 (<0.001)**	—	
HADS-D	0.035 (0.544)	**0.268 (<0.001)**	**0.568 (<0.001)**	—

Values are presented as Pearson’s correlation coefficients (*r*), with *p* values in parentheses. 25(OH)D = 25-hydroxyvitamin D; ESS = Epworth Sleepiness Scale; HADS-A = Hospital Anxiety and Depression Scale–Anxiety subscale; HADS-D = Hospital Anxiety and Depression Scale–Depression subscale. Statistically significant correlations (*p* < 0.05) are shown in bold.

**Table 6:** Univariable and Multivariable Logistic Regression Analyses of Factors Associated with Study Outcomes.

Outcome/Predictor	OR (95% CI)	*p*	aOR (95% CI)	*p*
**Vitamin D deficiency (<20 ng/mL)**				
Age (per 1-year increase)	0.902 (0.864–0.941)	**<0.001**	0.915 (0.873–0.959)	**<0.001**
Marital status: Not married (ref: Married)	3.195 (1.489–6.856)	**0.003**	1.654 (0.605–4.523)	0.327
Household: No child at home (ref: Child at home)	2.760 (1.419–5.368)	**0.003**	1.136 (0.451–2.861)	0.787
Night shift work (ref: Day shift)	2.053 (1.068–3.948)	**0.031**	1.136 (0.541–2.389)	0.736
Body-covering clothing (ref. Seasonal clothing)	0.910 (0.495–1.675)	0.762	1.047 (0.548–2.002)	0.889
Previous vitamin D supplement use (ref:No)	0.477 (0.228–0.998)	**0.050**	0.576 (0.262–1.264)	0.169
Professional experience: <10 years (ref: ≥20 years)	5.071 (1.907–13.489)	**0.001**	-	**-**
Professional experience: 10–20 years (ref: ≥20 years)	1.531 (0.631–3.713)	0.346	-	-
**Excessive daytime sleepiness (ESS > 10)**				
Night shift work (ref: Day shift)	1.124 (0.696–1.814)	0.635	1.276 (0.739–2.203)	0.382
Professional experience: <10 years (ref: ≥20 years)	1.404 (0.535–3.682)	0.491	1.320 (0.465–3.743)	0.602
Professional experience: 10–20 years (ref: ≥20 years)	2.564 (0.979–6.713)	0.055	2.582 (0.930–7.163)	0.069
Anxiety symptoms (HADS-A > 10)	3.723 (2.195–6.314)	**<0.001**	2.795 (1.553–5.031)	**0.001**
Depressive symptoms (HADS-D > 7)	2.793 (1.697–4.595)	**<0.001**	1.887 (1.077–3.306)	**0.027**
BMI (per 1 kg/m^2^ increase)	1.043 (0.991–1.097)	0.105	1.032 (0.976–1.091)	0.269
Smoking (pack-years)	0.966 (0.901–1.036)	0.328	-	-
**Anxiety symptoms (HADS-A > 10)**				
Excessive daytime sleepiness (ESS > 10)	3.723 (2.195–6.314)	**<0.001**	2.807 (1.576–5.001)	**<0.001**
Depressive symptoms (HADS-D > 7)	8.500 (4.618–15.648)	**<0.001**	7.332 (3.937–13.657)	**<0.001**
**Depressive symptoms (HADS-D > 7)**				
Excessive daytime sleepiness (ESS > 10)	2.793 (1.697–4.595)	**<0.001**	1.879 (1.081–3.266)	**0.025**
Anxiety symptoms (HADS-A > 10)	8.500 (4.618–15.648)	**<0.001**	7.285 (3.906–13.587)	**<0.001**
Night shift work (ref: Day shift)	1.324 (0.846–2.073)	0.219	1.300 (0.784–2.158)	0.310
BMI (per 1 kg/m^2^ increase)	1.021 (0.973–1.072)	0.393	1.017 (0.961–1.076)	0.554
Smoking (pack-years)	0.932 (0.871–0.998)	**0.044**	**-**	**-**

Values are presented as odds ratios (ORs) from univariable analyses and adjusted odds ratios (aORs) from multivariable analyses, with 95% confidence intervals (CIs). ESS = Epworth Sleepiness Scale; HADS = Hospital Anxiety and Depression Scale; BMI = body mass index. Vitamin D deficiency was defined as serum 25-hydroxyvitamin D [25(OH)D] < 20 ng/mL; excessive daytime sleepiness as ESS > 10; anxiety symptoms as HADS-A > 10; and depressive symptoms as HADS-D > 7. Not married included single, separated/divorced, and widowed participants. Reference categories are indicated in parentheses. A dash (-) indicates that the variable was not included in the corresponding multivariable model. Statistically significant *p* values (*p* < 0.05) are shown in bold.

## Data Availability

The data presented in this study are available on reasonable request from the corresponding author. The data are not publicly available due to ethical and privacy restrictions related to participant confidentiality.

## Abbreviations

25(OH)D	25-hydroxyvitamin D
BMI	Body mass index
CI	Confidence interval
DSM-5	Diagnostic and Statistical Manual of Mental Disorders, Fifth Edition
EDS	Excessive daytime sleepiness
ESS	Epworth Sleepiness Scale
HADS	Hospital Anxiety and Depression Scale
HADS-A	Hospital Anxiety and Depression Scale–Anxiety subscale
HADS-D	Hospital Anxiety and Depression Scale–Depression subscale
HPA	Hypothalamic–pituitary–adrenal
ICU	Intensive care unit
ICD-10	International Classification of Diseases, Tenth Revision
ICSD-3	International Classification of Sleep Disorders, Third Edition
IQR	Interquartile range
OR	Odds ratio
SWSD	Shift work sleep disorder

## References

[1] Geniş B., Coşar B., Taner M.E. (2020). Factors affecting mental status and effects of shift work system in healthcare workers. J. Psychiatr. Nurs..

[2] Books C., Coody L.C., Kauffman R., Abraham S. (2020). Night shift work and its health effects on nurses. Health Care Manag..

[3] Sun S.Y., Chen G.H. (2022). Treatment of circadian rhythm sleep–wake disorders. Curr. Neuropharmacol..

[4] American Psychiatric Association (2013). Diagnostic and Statistical Manual of Mental Disorders.

[5] Pallesen S., Bjorvatn B., Waage S., Harris A., Sagoe D. (2021). Prevalence of shift work disorder: A systematic review and meta-analysis. Front. Psychol..

[6] Selvi Y., Kandeğer A., Sayın A.A. (2016). Excessive daytime sleepiness. Curr. Approaches Psychiatry..

[7] Culpepper L. (2010). The social and economic burden of shift-work disorder. J. Fam. Pract..

[8] Turkish Society of Endocrinology and Metabolism (2025). Osteoporoz ve Metabolik Kemik Hastalıkları Tanı ve Tedavi Kılavuzu [Guideline for Diagnosis and Treatment of Osteoporosis and Metabolic Bone Diseases].

[9] Varkal M.A., Yıldız İ., Saygılı S., Yıldız M., Kılıç A., Darendeliler F.F., Oğuz F., Ünüvar E. (2015). One of the risk groups for vitamin D deficiency: Health workers. J. Istanb. Fac. Med..

[10] Lehnert M., Beine A., Burek K., Putzke S., Schlösser S., Pallapies D., Brüning T., Behrens T., Rabstein S. (2018). Vitamin D supply in shift working nurses. Chronobiol. Int..

[11] Pludowski P., Holick M.F., Grant W.B., Konstantynowicz J., Mascarenhas M.R., Haq A., Povoroznyuk V., Balatska N., Barbosa A.P., Karonova T. (2018). Vitamin D supplementation guidelines. J. Steroid Biochem. Mol. Biol..

[12] Goswami C., Law S., Zhang H., Park H., Maheta B., Arnott K., Hsu M., Truong K., Sheffield M., Iqbal Z. (2025). Are there prophylactic effects of vitamin D among healthier adult patients? A systematic review of randomized controlled trials. BMC Nutr..

[13] Humble M.B. (2010). Vitamin D, light and mental health. J. Photochem. Photobiol. B.

[14] Akpınar Ş., Karadağ M.G. (2022). Is vitamin D important in anxiety or depression? What is the truth?. Curr. Nutr. Rep..

[15] Alreshidi S.M., Rayani A.M. (2023). The correlation between night shift work schedules, sleep quality, and depression symptoms. Neuropsychiatr. Dis. Treat..

[16] Øyane N.M.F., Pallesen S., Moen B.E., Åkerstedt T., Bjorvatn B. (2013). Associations between night work and anxiety, depression, insomnia, sleepiness and fatigue in a sample of Norwegian nurses. PLoS ONE.

[17] Roman P., Perez-Cayuela I., Gil-Hernández E., Rodriguez-Arrastia M., Aparicio-Mota A., Ropero-Padilla C., Rueda-Ruzafa L. (2023). Influence of shift work on the health of nursing professionals. J. Pers. Med..

[18] Johns M.W. (1991). A new method for measuring daytime sleepiness: The Epworth Sleepiness Scale. Sleep.

[19] Ağargün M.Y., Çilli A.S., Kara H., Bilici M., Telcioğlu M., Semiz Ü.B., Başoğlu C. (1999). Validity and reliability of the Epworth Sleepiness Scale. Turk. Psikiyatr. Derg..

[20] Zigmond A.S., Snaith R.P. (1983). The Hospital Anxiety and Depression Scale. Acta Psychiatr. Scand..

[21] Aydemir Ö., Güvenir T., Küey L., Kültür S. (1997). Validity and reliability of the Turkish version of the Hospital Anxiety and Depression Scale. Turk. Psikiyatr. Derg..

[22] Holick M.F., Binkley N.C., Bischoff-Ferrari H.A., Gordon C.M., Hanley D.A., Heaney R.P., Murad M.H., Weaver C.M. (2011). Evaluation, treatment, and prevention of vitamin D deficiency: An Endocrine Society clinical practice guideline. J. Clin. Endocrinol. Metab..

[23] Vasileiou E., Nena E., Ntolios P., Zissimopoulos A., Constantinides T. (2026). Vitamin D levels, general health status, and work productivity among healthcare workers—A scoping review of published literature (2010–2025). Front. Nutr..

[24] Rizza S., Pietroiusti A., Farcomeni A., Mina G.G., Caruso M., Virgilio M., Magrini A., Federici M., Coppeta L. (2020). Monthly fluctuations in 25-hydroxy-vitamin D levels in day and rotating night shift hospital workers. J. Endocrinol. Investig..

[25] Tang H.Y., Ko W.S., Yan Y.H., Yu S.C., Chiou Y.L. (2022). Relationship between serum 25-hydroxyvitamin D [25(OH)D] levels and mental health in shift female nurses. Sci. Rep..

[26] Lee H.J., Choi H., Yoon I.Y. (2020). Impacts of serum vitamin D levels on sleep and daytime sleepiness according to working conditions. J. Clin. Sleep Med..

[27] Martelli M., Salvio G., Santarelli L., Bracci M. (2022). Shift work and serum vitamin D levels: A systematic review and meta-analysis. Int. J. Environ. Res. Public Health.

[28] Rotter M., Brandmaier S., Covic M., Burek K., Hertel J., Troll M., Bader E., Adam J., Prehn C., Rathkolb B. (2018). Night shift work affects urine metabolite profiles of nurses with early chronotype. Metabolites.

[29] Ungur A.P., Socaciu A.I., Barsan M., Rajnoveanu A.G., Ionut R., Socaciu C., Procopciuc L.M. (2025). Urine metabolomic patterns to discriminate the burnout levels and night-shift-related stress in healthcare professionals. Metabolites.

[30] Aykal G., Cerit N., Tekeli S.Ö., Ellidağ H.Y., Yılmaz N. (2016). Prevalence of vitamin D insufficiency and deficiency in operating room personel. Turk. Klin. Biyokim. Derg..

[31] Akçay D., Yıldırımlar A., Aksu S. (2021). Factors affecting sleep status of nurses. Turk. Klin. J. Nurs. Sci..

[32] Arbour M., Tanner T., Hensley J., Beardsley J., Wika J., Garvan C. (2019). Factors that contribute to excessive sleepiness in midwives practicing in the United States. J. Midwifery Womens Health.

[33] Terauchi M., Ideno Y., Hayashi K. (2024). Effect of shift work on excessive daytime sleepiness in female nurses: Results from the Japan Nurses’ Health Study. Ind. Health.

[34] Alexandropoulou A., Vavougios G.D., Hatzoglou C., Gourgoulianis K.I., Zarogiannis S.G. (2019). Risk assessment for self-reported obstructive sleep apnea and excessive daytime sleepiness in a Greek nursing staff population. Medicina.

[35] Carvalho V.P., Barcelos K.A., Oliveira E.P., Marins S.N., Rocha I.B.S., Sousa D.F.M., Moreira B.C., Almeida G.A., Carneiro M.L.S., Silva J.D.F. (2021). Poor sleep quality and daytime sleepiness in health professionals: Prevalence and associated factors. Int. J. Environ. Res. Public Health.

[36] Uz B., Savaşan E., Soğancı D. (2022). Anxiety, depression and burnout levels of Turkish healthcare workers at the end of the first period of COVID-19 pandemic in Turkey. Clin. Psychopharmacol. Neurosci..

[37] Chen L., Luo C., Liu S., Chen W., Liu Y., Li Y., Du Y., Zou H., Pan J. (2019). Excessive daytime sleepiness in general hospital nurses: Prevalence, correlates, and its association with adverse events. Sleep Breath..

[38] Ezin A.A., Ekşioğlu Z.Ş., Güven R. (2018). Measurement of anxiety and depression level for nurses. Bitlis Eren Univ. J. Sci. Technol..

[39] Dai C., Qiu H., Huang Q., Hu P., Hong X., Tu J., Xie Q., Li H., Ren W., Ni S. (2019). The effect of night shift on sleep quality and depressive symptoms among Chinese nurses. Neuropsychiatr. Dis. Treat..

[40] Musazadeh V., Keramati M., Ghalichi F., Kavyani Z., Ghoreishi Z., Alras K.A., Albadawi N., Salem A., Albadawi M.I., Salem R. (2023). Vitamin D protects against depression: Evidence from an umbrella meta-analysis on interventional and observational meta-analyses. Pharmacol. Res..

[41] Karim S., Natche J., Babli S.A., Avinash, Matakhov D., Ferdous S., Jeyakkodi H., Alamu O.S., Rizwanullah U.F.N., Hameed S.A. (2026). Association between low vitamin D levels and depression: A systematic review and meta-analysis. Cureus.

